# THE FEASIBILITY AND ACCEPTABILITY OF PERTURBATION BALANCE TRAINING IN RURAL COMMUNITIES: A MIXED METHODS STUDY

**DOI:** 10.1093/geroni/igad104.1956

**Published:** 2023-12-21

**Authors:** Justin Whitten, David Graham, Michelle Grocke, Bryant O’Leary, Julie Riley, Dawn Tarabochia

**Affiliations:** Montana State University, Bozeman, Montana, United States; Montana State University, Bozeman, Montana, United States; Montana State University, Bozeman, Montana, United States; Montana State University, Missoula, Montana, United States; Montana State University, Broadus, Montana, United States; Montana State University, Bozeman, Montana, United States

## Abstract

Falls are a leading cause of morbidity and mortality in older adults. Older adults in rural communities have an elevated risk of falling. Perturbation-Based Balance Training (PBT) is an effective fall-prevention paradigm. However, it’s acceptability and feasibility in community settings is unknown. The purpose of this study was to evaluate the acceptability and feasibility of a community-based PBT program to local community-dwelling older adults and practitioners. Nineteen healthy older adults (60+ years) completed a 6-week PBT program, implemented in conjunction with three community practitioners. Measures of balance, balance confidence and fear of falling were assessed before and after training. Feasibility and acceptability were assessed prospectively, concurrently, and retrospectively via field notes, surveys, and interviews within the Theoretical Framework of Acceptability. PBT was perceived as effective by older adults. Perceived increases in balance awareness, low burden/opportunity-cost and enjoyment of novel training were identified as facilitators. Notably, participants with poor balance tended to perceive PBT as less acceptable and demonstrated apprehensive and avoidant behavior, reinforcing the importance of comprehensive screening prior to participation in PBT. Practitioners perceived PBT as acceptable, with efficacy, low burden/opportunity-cost, and ethicality being primary facilitators. While the portable nature of the perturbation treadmill increased feasibility in rural communities, cost of equipment and transportation were perceived as substantial barriers to implementation. These results suggest PBT is acceptable in rural communities and demonstrate a need for training modalities that are more feasibly implemented in a community setting. Further investigation of low-tech alternatives such as manual PBT may offer potential solutions.

